# Engineering DNA Backbone Interactions Results in TALE Scaffolds with Enhanced 5-Methylcytosine Selectivity

**DOI:** 10.1038/s41598-017-15361-1

**Published:** 2017-11-08

**Authors:** Preeti Rathi, Anna Witte, Daniel Summerer

**Affiliations:** Department of Chemistry and Chemical Biology, Technical University of Dortmund, Otto-Hahn-Str. 4a, 44227, Dortmund, Germany

## Abstract

Transcription activator-like effectors (TALEs) are DNA major-groove binding proteins widely used for genome targeting. TALEs contain an N-terminal region (NTR) and a central repeat domain (CRD). Repeats of the CRD selectively recognize each one DNA nucleobase, offering programmability. Moreover, repeats with selectivity for 5-methylcytosine (5mC) and its oxidized derivatives can be designed for analytical applications. However, both TALE domains also nonspecifically interact with DNA phosphates via basic amino acids. To enhance the 5mC selectivity of TALEs, we aimed to decrease the nonselective binding energy of TALEs. We substituted basic amino acids with alanine in the NTR and identified TALE mutants with increased selectivity. We then analysed conserved, DNA phosphate-binding KQ diresidues in CRD repeats and identified further improved mutants. Combination of mutations in the NTR and CRD was highly synergetic and resulted in TALE scaffolds with up to 4.3-fold increased selectivity in genomic 5mC analysis via affinity enrichment. Moreover, transcriptional activation in HEK293T cells by a TALE-VP64 construct based on this scaffold design exhibited a 3.5-fold increased 5mC selectivity. This provides perspectives for improved 5mC analysis and for the 5mC-conditional control of TALE-based editing constructs *in vivo*.

## Introduction

**Figure 1:**
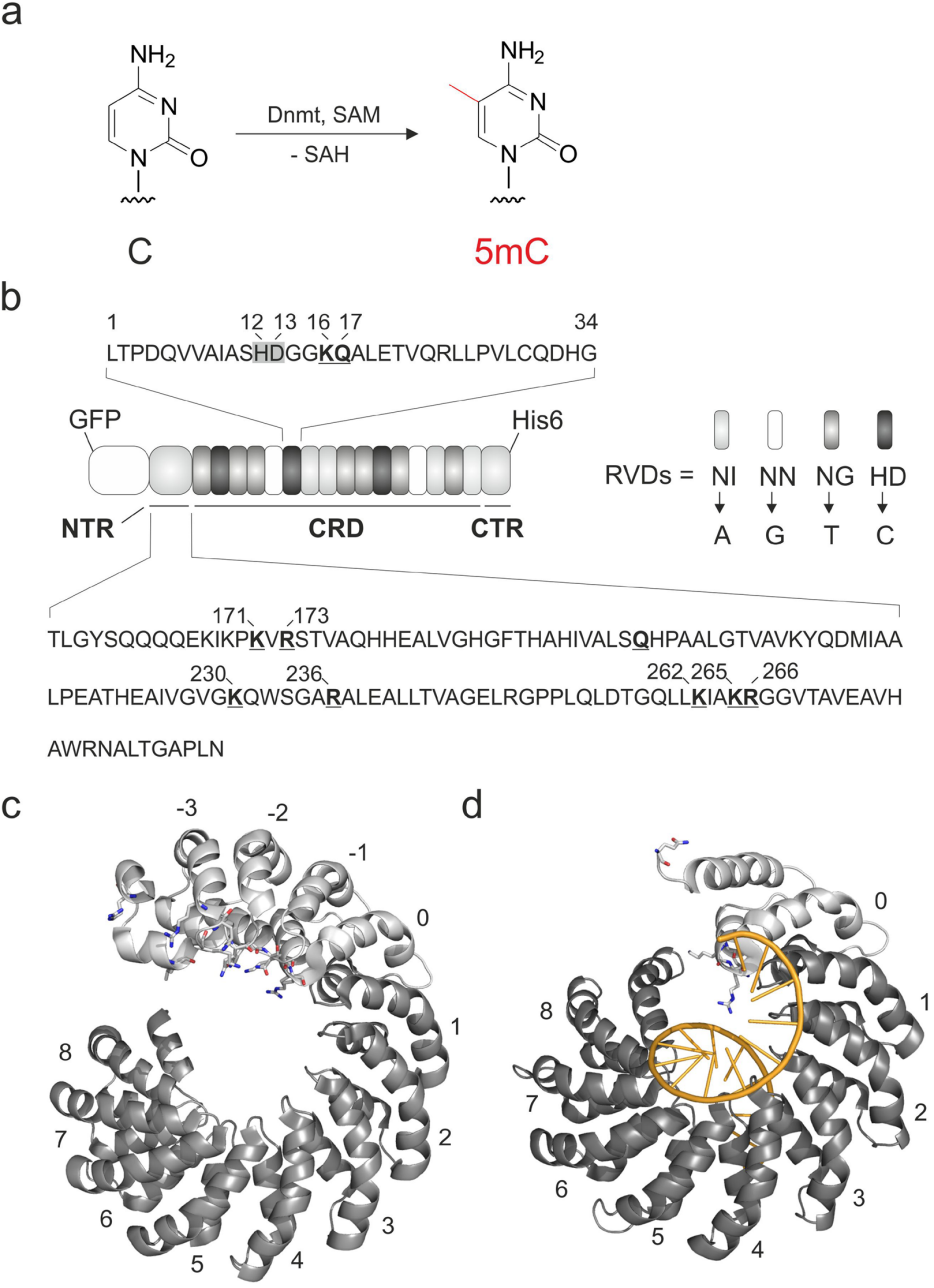
Cytosine 5-methylation, overall features of the TALE scaffold, and TALE amino acid positions engineered in this study to obtain TALE scaffolds with increased C/5mC selectivity. (**a**) Conversion of the nucleobase C into the epigenetic nucleobase 5mC by DNA methyltransferases (Dnmt) using S-adenosylmethionine (SAM) as a cofactor (SAH: S-adenosylhomocystein). (**b**) Cartoon showing the general features of employed TALEs. The amino acid sequence of one representative TALE repeat is shown on top, with RVD amino acids 12 and 13 marked with a grey box and KQ diresidue at positions 16 and 17 involved in DNA phosphate binding in bold and underlined. Amino acid sequence of the N-terminal region (NTR) is shown on the bottom with amino acids suspected to be involved in DNA phosphate binding in bold and underlined. TALE repeats with selectivity for canonical nucleobases are shown on the right with RVDs specified. CRD: central repeat domain. CTR: C-terminal region. (**c**) Crystal structure of TALE in DNA-unbound state (pdb entry 4HPZ)^40^. Eight regular repeats of the CRD (dark grey, numbering 1–8) and repeats of the NTR (light grey) are shown. Repeats are numbered in relation to the T-binding 0 repeat. Amino acids suspected to be involved in DNA phosphate binding are shown as sticks. (**d**) Crystal structure of TALE in DNA-bound state (pdb entry 3V6T)^33^; (DNA shown as orange cartoon) with features marked as in Fig. 1c.

5-methylcytosine (5mC) is an epigenetic DNA nucleobase that acts as a regulatory element of human gene expression with important roles in development and diseases^1,2^. 5mC is introduced into DNA by DNA methyltransferases (Dnmt) that use S-adenosylmethionine (SAM) as methyl-donating co-factor (Fig. 1a). Moreover, ten-eleven translocation (TET) enzymes can oxidize 5mC to the three additional derivatives 5-hydroxymethylcytosine (5hmC), 5-formylcytosine (5fC) and 5-carboxylcytosine (5caC)^3–7^ in a step-wise fashion. These nucleobases are intermediates of an active demethylation pathway^8,9^, establishing 5mC-directed gene expression regulation as a dynamic process.

Further studies on the involvement of 5mC in development and disease require simple methods for its analysis in genomic DNA sequences of interest. C and 5mC can be distinguished by selective deamination with bisulfite followed by sequence analysis^10^, and this approach can be combined with additional redox or tagging reactions to analyse oxidized derivatives of 5mC^11–15^. Alternative analytical approaches rely on the selectivity of restriction endonucleases, antibodies, methyl-CpG-binding domains (MBD) or tagging reactions combined with (bisulfite) sequencing, or with affinity enrichment for global isolation of all modified DNA followed by sequence analysis^9,16^. Another group of emerging approaches relies on proteins that provide direct nucleobase recognition selectivity in the context of both canonical and epigenetic nucleobases^17^ and can be employed in single molecule sequencing, such as nanopores^18–20^ and DNA polymerases^21–23^.

We have recently reported the use of engineered transcription activator-like effectors (TALEs^24,25^) as programmable DNA binding domains for the analysis of 5mC and other epigenetic cytosine 5-modifications^26,27^. TALEs consist of an N-terminal region (NTR), a C-terminal region (CTR), and a central domain of regular repeats (CRD) that each selectively recognizes one nucleobase via one of two variable amino acids (repeat variable diresidue, RVD). This recognition occurs via a predictable code with the RVDs NI, NN, NG and HD (amino acid positions 12 and 13 within each TALE repeat) preferentially binding A, G(A), T, and C, respectively^28,29^ (see Fig. 1b, alternative RVDs have been identified^30–32^). Recognition occurs via the major groove^33,34^ that displays unique chemical information not only for each canonical base pair, but also for each epigenetic cytosine nucleobase^35^. This enables the design of specific TALE repeat codes for epigenetic nucleobases^36–38^, and consequently for their direct and simple analysis at user-defined genomic sites by affinity enrichment^39^. The most critical property of TALEs in view of such applications is the epigenetic nucleobase selectivity, since it defines the dynamic range of the assay. However, though engineering of the direct interaction between TALE repeat and nucleobase is a successful strategy to increase this selectivity^36–38^, this interaction is highly confined in natural TALE repeats, resulting in a limited structural space for engineering. Alternative strategies for increasing this selectivity are thus highly desired.

Besides direct interactions with the DNA nucleobases, TALEs recognize the DNA backbone via multiple interactions throughout the scaffold: crystal structures of DNA-unbound TALEs have shown that the NTR consists of four cryptic repeats (Fig. 1c, repeat numbering −3–0)^40^. These bear a large number of basic residues that line its putative DNA binding surface and undergo or are suspected to undergo DNA phosphate interactions (Fig. 1c,d). Moreover, each of the repeats within the CRD bears a GG diresidue at positions 14 and 15 and a KQ diresidue at positions 16 and 17 that both interact with the backbone phosphate (see below for details)^33,34^. Finally, the CTR contains multiple basic residues involved in DNA binding^41^.

We hypothesized that deletion of selected TALE-DNA-phosphate interactions may lower the overall contribution of non-nucleobase-specific binding energy to complex formation and as a consequence to increase the contribution of nucleobase-selective binding energy. This may result in enhanced 5mC-selectivity of TALEs due to larger relative differences in binding energy between C- and 5mC-containing target sequences. To this end, we report a broad evaluation of basic residues in both the NTR and CRD in view of their involvement in binding to DNA sequences containing single C versus 5mC positions. Using alanine mutagenesis^42^, we first studied a group of basic residues in the NTR and identified several TALE mutants with increased 5mC selectivity. We then analysed the KQ diresidue of the regular TALE repeats in single and multiple TALE repeat positions, and identified further improved mutants. Combination of mutations in the NTR and in single CRD repeats was highly synergetic and resulted in novel TALE scaffolds for simple, affinity-enrichment based 5mC analysis at single, user-defined genomic positions with increased selectivity. Moreover, the scaffold design enabled increased 5mC-selectivity of TALE-based transcription activation in HEK293T cells, providing new perspectives for 5mC analysis and 5mC-conditional control of TALE-based editing constructs *in vivo*.

## Materials and Methods

### Construction of mutant TALE expression plasmids

The following TALE entry plasmids bearing N-terminal mutations were created by Quikchange site directed mutagenesis (Agilent): pPrR766 (K171A), pPrR767 (R173A), pPrR768 (R236A), pPrR769 (R266A), pPrR770 (K171A&R173A), pPrR771 (K262A) and pPrR772 (K262A&K265A&R266A). Mutagenesis reactions contained 10 ng plasmid, 0.4 µM each primer, 1x Pfu polymerase buffer and 2 units of Pfu polymerase in a final reaction volume of 25 µL. Reactions were performed on plasmid pAnI521 using the primer pairs oPrR1918/oPrR1919, oPrR1920/oPrR1921, oPrR1922/oPrR1923, oPrR1924/oPrR1925, oPrR1926/oPrR1927 or oPrR1928/oPrR1929 and on plasmid pPrR772 using primer pair oPrR1930/oPrR1931. Reactions were incubated at 95 °C for 30 sec followed by 20 cycles of 95 °C for 30 sec, 55 °C for 30 sec, followed by 68 °C for 8 min. After addition of 1 µL of *DpnI* endonuclease (NEB), the reactions were incubated at 37 °C for 1 h and at 80 °C for 20 min. TALEs were assembled according to a previously published protocol^43^ using entry plasmids pAnI521, pPrR766, pPrR767, pPrR768, pPrR769, pPrR770, pPrR771, pPrR772 or pPrR773 in golden gate 2 reactions, resulting in plasmids coding for the respective TALE proteins with N-terminal GFP tag and a C-terminal His6 tag (for protein sequences, see SI Fig. 1).

### Expression and purification of TALE proteins

TALE proteins were expressed and purified as described previously^44^. Briefly, single clones of *E. coli* BL21(DE3) Gold transformed with a TALE expression plasmid were grown in LB media supplemented with 50 µg/ml carbenicillin at 37 °C overnight. These cultures were diluted 50-fold into LB medium supplemented with the same antibiotic and were incubated at 37 °C and 200 rpm shaking until an OD_600_ of ~0.4 was reached. IPTG was added to a concentration of 0.2 mM and the cultures were harvested by centrifugation after 5 h of further incubation under the same conditions. The pellets were lysed in Lysis-buffer (10 mM Tris-HCl, 300 mM NaCl, 2.5 mM MgCl_2_, 0.1% Triton X-100, pH = 9) containing 1 mM PMSF and 50 µg/ml lysozyme (Sigma Aldrich) by shaking at room temperature at 1400 rpm for 30 min. The suspension was pelleted by centrifugation, the supernatant was collected and extracted with Ni-NTA (Thermo Scientific). Ni-NTA was washed two times with 4 x PBS-Buffer (0.55 M NaCl, 43 mM KCl, 69 mM Na_2_HPO_4_·2H_2_O, 24 mM KH_2_PO_4_, pH = 8), four times with wash buffer (50 mM NaH_2_PO_4_·H_2_O and 300 mM NaCl pH = 8) containing 20 mM imidazole and once with wash buffer containing 50 mM imidazole. The protein was eluted three times with wash buffer containing 500 mM imidazole. Pooled elution fractions were added to a dialysis tube (Carl Roth) and dialyzed against TALE Storage Buffer (20 mM Tris-HCl pH = 7.5, 200 mM NaCl, 10% Glycerole, 1 mM DTT). Purity of the TALE protein was analysed on SDS PAGE stained with Gelcode blue (Thermo Scientific) and quantified by a BCA assay (Pierce). The proteins were snap-frozen and stored in aliquots at −80 °C in TALE storage buffer including 0.1 mg/ml bovine serum albumin (BSA, NEB).

### Electromobility shift assays

Oligonucleotide pairs oGrK1591/oGrK1592 and oGrK1617 or oPrR2121/oPrR2123 and oPrR2122 were hybridized in 1x BGrK2 buffer (20 mM Tris, 50 mM NaCl, 5 mM MgCl_2_ and 5% v/v glycerol, pH = 8) by incubating at 95 °C for 5 min and at RT for 30 min. Then, 0.5 pmol of the respective DNA duplex was incubated with 0.1 pmol of the respective TALE protein in 1x BGrK2 buffer with a final volume of 10 µL. The reaction was kept at room temperature for 30 min and then on ice for 30 min. The EMSA gel (0.5x TAE buffer, 8% Rotipherese gel 40 (Carl-Roth), 0.1% APS and 0.01% TEMED) was pre-run at 4 °C for 30 min in a Mini Protean vertical electrophoresis cell (Bio-Rad), loaded with 5 µL sample and then run for 90 min at 4 °C and 70 V. GFP fluorescence of the gels was recorded with a Typhoon FLA-9500 laser scanner (GE healthcare) and analysed using the software Image J. TALE-DNA complex formation was calculated by building the ratios of the shifted band (TALE-DNA complex) and the complete area of the same lane.

### Preparation of genomic DNA samples

Human gDNA (male Yoruban individual, Encode sample NA18507) was obtained from the Coriell Institute. For erasing epigenetic cytosine modifications, gDNA samples were whole genome amplified using the REPLI-g mini kit (Qiagen) and purified using the QIAamp DNA mini kit (Qiagen). gDNA samples were sheared by sonication (Branson Sonifier 250, 20% power, 20 cycles of 30 s on/30 s off), resulting in a fragment size distribution between 100–1000 bp (SI Fig. 3). A fraction of the fragmented gDNA samples was methylated using 20 U *M.Sss*I DNA methyltransferase (NEB) in 200 μl Buffer NEB2 (50 mM NaCl, 10 mM Tris-HCl, 10 mM MgCl_2_, 1 mM DTT, pH = 7.9) supplemented with 640 μM S-adenosylmethionine (SAM) by incubation at 37 °C for 14 h (after 4 h, additional 4 μl of 32 mM SAM were added) and purified using the QIAamp DNA mini kit (Qiagen). Fractions of both non-methylated and methylated gDNA were bisulfite-converted with the EpiTect Bisulfite Kit (Qiagen), a representative target region was amplified and sequenced, confirming both the absence and presence of CpG methylation in the gDNA target before and after *M.SssI* methylation, respectively (SI Fig. 4).

### Genomic 5mC analysis by affinity enrichment with TALEs

Affinity enrichment experiments were conducted similar to a previously reported protocol^39^. Briefly, Ni-NTA magnetic agarose beads (Qiagen) were freshly washed/equilibrated before each enrichment with buffer X (150 mM NaCl, 30 mM Tris-HCl, 5 mM MgCl_2_, 0.5 mg/ml BSA, pH = 7.9) at 4 °C using a 12-tube magnetic stand (Qiagen) with keeping the overall volume constant. For enrichment, 2 µL (100 ng/µL) of gDNA, 8 µL (4 µM) TALE protein and 1.2 µL 10x buffer X (with 0.5 mM DTT added freshly) were added to 0.8 µL nuclease-free water. The mixture was incubated on ice for 60 min. Afterwards, 483 µL of buffer X (with 0.5 mM DTT added freshly) and 5 µL of equilibrated beads were added and the samples were incubated at 4 °C and 700 rpm shaking for 60 min in a thermomixer (Eppendorf). The tubes were placed on the magnetic stand for 2 min and the liquid was carefully removed without disturbing the beads. The beads were washed once with 500 µL of pre-chilled buffer X (with 0.5 mM DTT added freshly) by slowly pipetting up and down once and the tubes were placed on the magnetic stand for 2 min. The liquid was completely removed and 500 µL of nuclease-free water was added. The tube was shaken at 1400 rpm at 95 °C for 5 min. The tubes were cooled down to room temperature, spinned shortly (2–3 sec) and placed on the stand. The supernatant was collected in a fresh tube, dried in a speed-vac and re-suspended with 50 µL of nuclease-free water. qPCRs were conducted with 12.5 µL GoTaq qPCR Master Mix (Promega), 5 µL of primer pair (1 µM each, for oligonucleotide sequences, see the SI) and 7.5 µL of the sample as template. Copy number quantification for enriched gDNA was done by linear regression from serial dilutions of the respective gDNA with each primer pair. Original and enzymatically methylated gDNA did not differ significantly in qPCR efficiency.

### TALE-based *in vivo* transcription activation with 5mC-selectivity

Plasmids pPrR814synC and pPrR814synmC bearing a single C or mC in the TALE binding site were constructed as follows: Oligo pairs oAnW2016/oAnW2017 and oPrR2248/oAnW2017 were hybridized (4 µM each) in 1x Cut-Smart buffer (NEB) by heating at 95 °C for 5 min and incubating at RT for 30 min. Plasmid pAnW755 and the hybridized oligonucleotide duplexes containing or not containing the single mC position were digested with each 10 units of *SalI* HF and *SpeI* (NEB) and purified using a PCR purification kit (Thermo Scientific). Plasmids p814synC and p814synmC were constructed by ligation of the digested pAnW755 with the digested oligonucleotide duplexes using T4 DNA ligase. Ligation reactions were purified via PCR purification and quantified by UV absorption measurement at 260 nm (Nanodrop 2000, Thermo Scientific). HEK293T cells were maintained in DMEM (PAN) media supplemented with 1% penicillin/streptomycin, 10% FBS (PAN) and 1% L-Glutamine (PAN). 1.6 × 10^4^ cells were cultured in a 96 well plate overnight prior transfection. Opti MEM (Gibco) and lipofectamin 2000 (Thermo Fisher) were mixed according to the manufacturer’s protocol. 25 ng of plasmid from group A (encoding a TALE binding site and a minCMV promoter upstream of a firefly luciferase gene) and 175 ng of plasmid from group B (encoding the TALE-VP64 fusion constructs; for plasmid maps, see the SI Fig. 5) were pipetted in pairs as follows: pAnW755/pAnW818, pAnW814/pAnW818, pAnW814/pAnW893, pPrR814synC/p818, pPrR814synmC/pAnW818, pPrR814synC/pAnW893, pPrR814synmC/pAnW893, pPrR814synC/pPrR1149 and pPrR814synmC/pPrR1149. Transfection mix was added to each plasmid pair and incubated for 20 min at RT. The solution was added to wells of the 96 well plate and incubated at 37 °C and 5% CO_2_ for 48 hr. Each well was then washed with 20 µl of DPBS (PAN) and the supernatant removed. 40 µl of lysis buffer (100 mM NaH_2_PO_4_ and 0.2% Triton X-100) was added to each well and mixed vigorously. The plate was then incubated on ice for 20 min. After incubation, 20 µL of the lysis solution from each well was combined with each 90 µL of Bright-Glo (Promega) in a second 96 well plate. The plate was quickly spinned down and the luminescence was immediately measured on a TECAN M1000 plate reader (wavelength 380–600 nM). Ratio of luminescence from each sample to that of a sample transfected with TALE_9a_26(HD) and a nonmethylated luciferase reporter plasmid (that had not been ligated with a synthetic DNA oligonucleotide duplex) was plotted as relative luminescence. The error bars represent standard errors from triplicate experiments. For analysing the integrity of the synthetically introduced mC position in plasmids over the duration of the experiment, the samples were collected after 48 h of incubation, DNA was extracted with a QiaAmp DNA mini kit (Qiagen) and converted with bisulphite (Qiagen EpiTect kit). Nested PCR was performed on these samples with primer pairs oPrR2425/oPrR2426 followed by oPrR2424/oPrR2427 using the Epitect MSP kit (Qiagen). The PCR product was purified by agarose gel electrophoresis, extracted with a PCR purification kit (Thermo Scientific) and sequenced with primer oPrR2427 (for sequencing traces, see the SI Fig. 6).

## Results and Discussion

### Evaluation of basic amino acid residues in the N-terminal region

**Figure 2:**
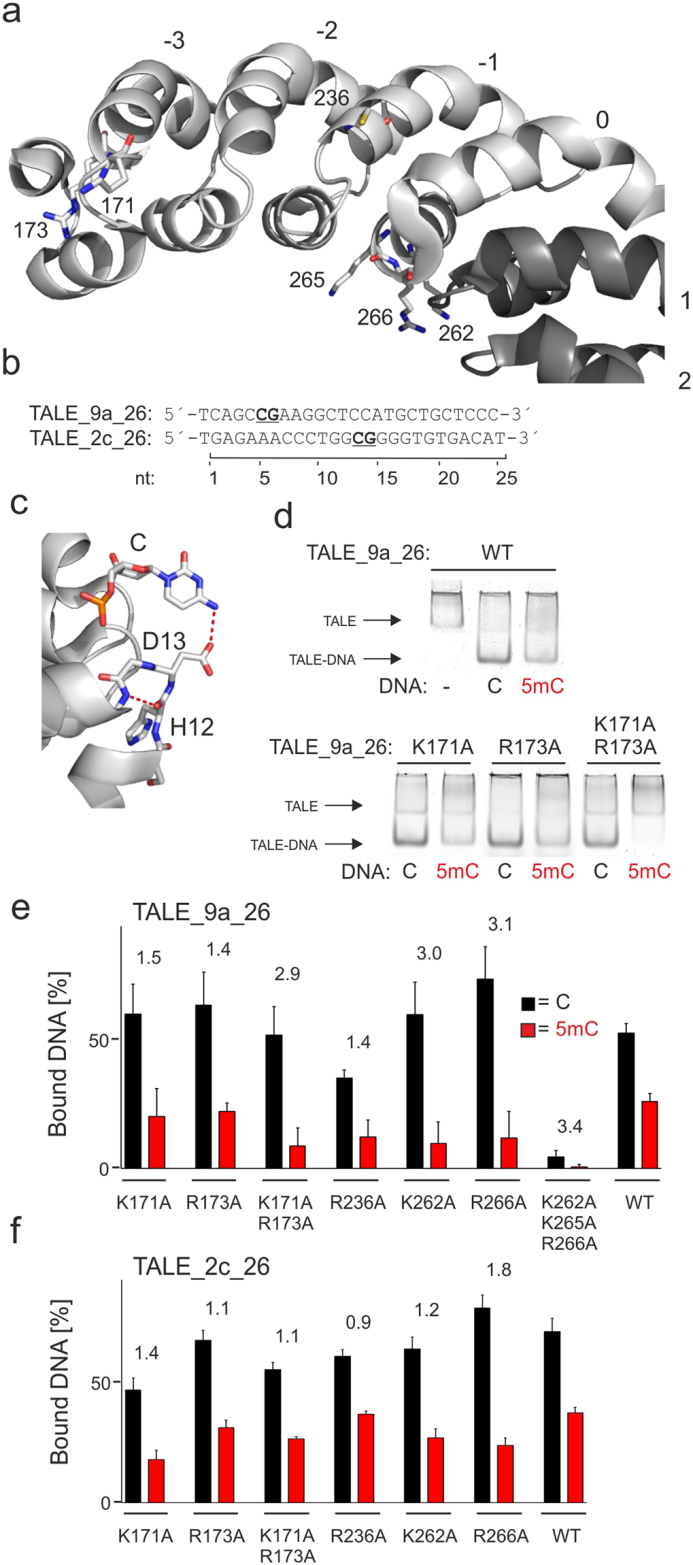
Influence of basic residues in the TALE NTR on affinity and C/5mC selectivity. (**a**) Crystal structure of the NTR of a TALE in DNA-unbound state (pdb entry 4HPZ)^40^. Repeats of the NTR are shown in light grey, repeats of the CRD in dark grey. Repeats are numbered as in Fig. 1c,d and amino acids suspected to be involved in DNA phosphate binding are shown as sticks. Note that this particular structure contains a R236C mutation. (**b**) Target sequences of employed TALEs with CpG dinucleotides containing the variable C/5mC position shown bold and underlined. (**c**) Interaction of RVD HD (amino acids 12 and 13 of TALE repeat) with cytosine (C) in a crystal structure of a TALE-DNA complex (pdb entry 3V6T)^33^. Hydrogen bonds are shown as dotted red lines. (**d**) EMSA assay with TALE_9a_26 WT and selected NTR alanine mutants using DNA duplexes with sequence shown in Fig. 2b and a single C or 5mC at position 5. Lane 1 and Lane 2/3 of the WT gel are individually cropped from the same gel with same exposure. Lanes of the TALE_9a_26 gel are from the same gel and not individually cropped. (**e**) Quantification of triplicate EMSA assays with TALE_9a_26 and NTR alanine mutants as shown. Data for DNA containing a single C at position 5 are shown in black, and ones containing a single 5mC are shown in red. Fold change of selectivity over that of WT TALE_9a_26 is indicated for each TALE above columns. (**f**) Quantification of triplicate EMSA assays with TALE_2c_26 and NTR alanine mutants. Fold change of selectivity over that of WT TALE_2c_26 is indicated for each TALE above columns.

Both NTR and CTR of TALEs contain basic lysine (K) and arginine (R) residues with suspected or proven involvement in DNA binding^33,34,40^. However, though the full-length CTR of several TALE scaffolds contains multiple of such residues and a high positive net charge, we previously employed TALE scaffolds for 5mC detection that contained a truncated 23 amino acid CTR with only a single basic residue and no positive net charge^39^. We thus did not study the CTR, but focused on the influence of basic residues in the four cryptic NTR repeats on DNA binding affinity and C/5mC selectivity. Crystal structures of a DNA-unbound TALE have shown that each of the four cryptic NTR repeats contains basic residues at positions that suggest backbone interactions in the DNA-bound state^40^ (Figs. 1b,c and 2a show sequence position and structures of residues targeted in this study). The involvement of these residues in DNA binding is further suggested by the findings that a truncated 140 amino acid NTR domain without CRD and CTR is capable of binding to dsDNA in a sequence-independent manner^40^ and that TALEs require at least a minimal NTR fragment to reach full activity in the form of different fusion constructs^45–48^. However, single mutations of these residues to noncharged residues showed little effect on DNA binding in isothermal calorimetry (ITC) assays, whereas mutagenesis of multiple residues strongly decreased DNA binding^40^. Interestingly, glutamine mutations of at least three basic residues in the NTR of TALE nucleases (TALEN), though strongly reducing activity, led to increased canonical sequence selectivity in a TALEN selection protocol^41^.

To gain insights into the involvement of basic NTR residues in epigenetic nucleobase selectivity of TALEs, we constructed TALEs targeting a 26 nt sequence in human chromosome 9 (chr9:21,974,786–21,974,811) with or without alanine mutations at single or multiple basic residues in the NTR (TALE_9a_26 Fig. 2b). We constructed TALEs based on a *Xanthomonas axonopodis* scaffold^45^ by the golden gate assembly protocol^43^. TALEs had an N-terminal green fluorescent protein (GFP)-domain, a shortened, *AvrBs3*-type TALE NTR (+136 instead of +288 amino acids starting from canonical repeat 1) and a C-terminal His6 tag (for protein sequences, see SI Fig. 1). We expressed TALEs in *E. coli* and purified them by Ni-NTA chromatography (SI Fig. 2). We employed these TALEs in electromobility shift assays (EMSA) using the GFP domain for signal readout together with a 5-fold molar excess of unlabeled DNA oligonucleotide duplexes containing the TALE_9a_26 sequence with either a C or a 5mC at nucleotide position 5 (Fig. 2b, the 5′-terminal T nucleotide is defined as position 0). We targeted this variable nucleotide position with a TALE repeat containing the natural RVD HD that is engaged in a hydrogen bond with the 4-amino group of the cytosine nucleobase (Fig. 2c). This interaction is sensitive to the presence of the 5-methyl group in 5mC, enabling the detection of single 5mC positions in large genomes via 5mC-dependent target sequence enrichment^39^. EMSA revealed the formation of single, defined TALE-DNA complexes with increased electromobility compared to the free TALE protein (Fig. 2d shows exemplary raw data). In this assay, the wild type (WT) TALE_9a_26 protein exhibited a moderate C over 5mC selectivity of 2.0-fold (note that TALE selectivities obtained from EMSA assays appear markedly lower than selectivities obtained from competitive assays or affinity enrichment experiments^39^, presumably as a result of their unique search mode and ability to stay physically associated with DNA even in absence of specific target sequences^49^).

The mutations K171A and R173A (both located in NTR repeat −3, Fig. 2a) only weakly affected DNA binding affinity for both C or 5mC, resulting in a 1.4–1.5-fold higher selectivity compared to the WT TALE in both cases. However, when introduced as double mutation, affinity to the 5mC containing DNA was markedly decreased, resulting in a 2.9-fold higher selectivity (Fig. 2e). We next introduced a R236A mutation in NTR repeat −1, since the crystal structure of a DNA-bound TALE shows that this residue makes a direct DNA phosphate interaction (not shown in structure of Fig. 2a that contains a R236C mutation)^34^. However, this resulted in decreased affinity for both C and 5mC and an only 1.4-fold higher selectivity than observed for WT TALE_9a_26 (Fig. 2e). Finally, we focussed on repeat 0 that is generally known to bind a T nucleobase at position 0 (Fig. 2b) of TALE target sequences via a stacking interaction with W232^33,34^. This repeat contains the three basic residues K262, K265 and R266 (Fig. 2a), of which K265 is engaged in a water-mediated DNA phosphate interaction and R266 in an unusual hydrogen bond to the N7 atom of a CRD-bound adenine in one crystal structure^34^. Interestingly, both a K262A and a R266A mutation resulted in a ~3-fold increased selectivity compared to the WT (Fig. 2e). In contrast, a K262A/K265A/R266A triple mutation - though still exhibiting selectivity - strongly decreased DNA binding, consistent with previous results^40,41^ (Fig. 2e).

To get insights into potential sequence- and 5mC-positional dependencies of the observed effects, we evaluated all mutations leading to functional TALEs in a second sequence context with an altered, more central C/5mC-variable position. In a TALE targeting a sequence in human chromosome 2 (chr2:98,330,240–98,330,265) with C or 5mC at position 13 (TALE_2c_26, Fig. 2b), we again observed only a weak (1.1- and 1.4-fold) increase in selectivity for the single mutations K171A and R173A, and the K171A/R173A double mutation did not lead to a further increase (Fig. 2f). The R236A mutation again did not positively affect selectivity, the K262A mutation only 1.2-fold. The highest increase was observed for the R266A mutation (1.8-fold). These data show that removal of specific basic residues in the NTR can result in TALE scaffolds with increased C versus 5mC selectivity, mainly due to a selectively decreased affinity to 5mC-containing DNA. This was observed for two unrelated sequence contexts with two different C/5mC-variable positions, with stronger increases for the TALE_9a_26 sequence and its more N-terminal C/5mC-variable position within the CRD.

### Evaluation of basic amino acid residues in the central repeat domain

**Figure 3:**
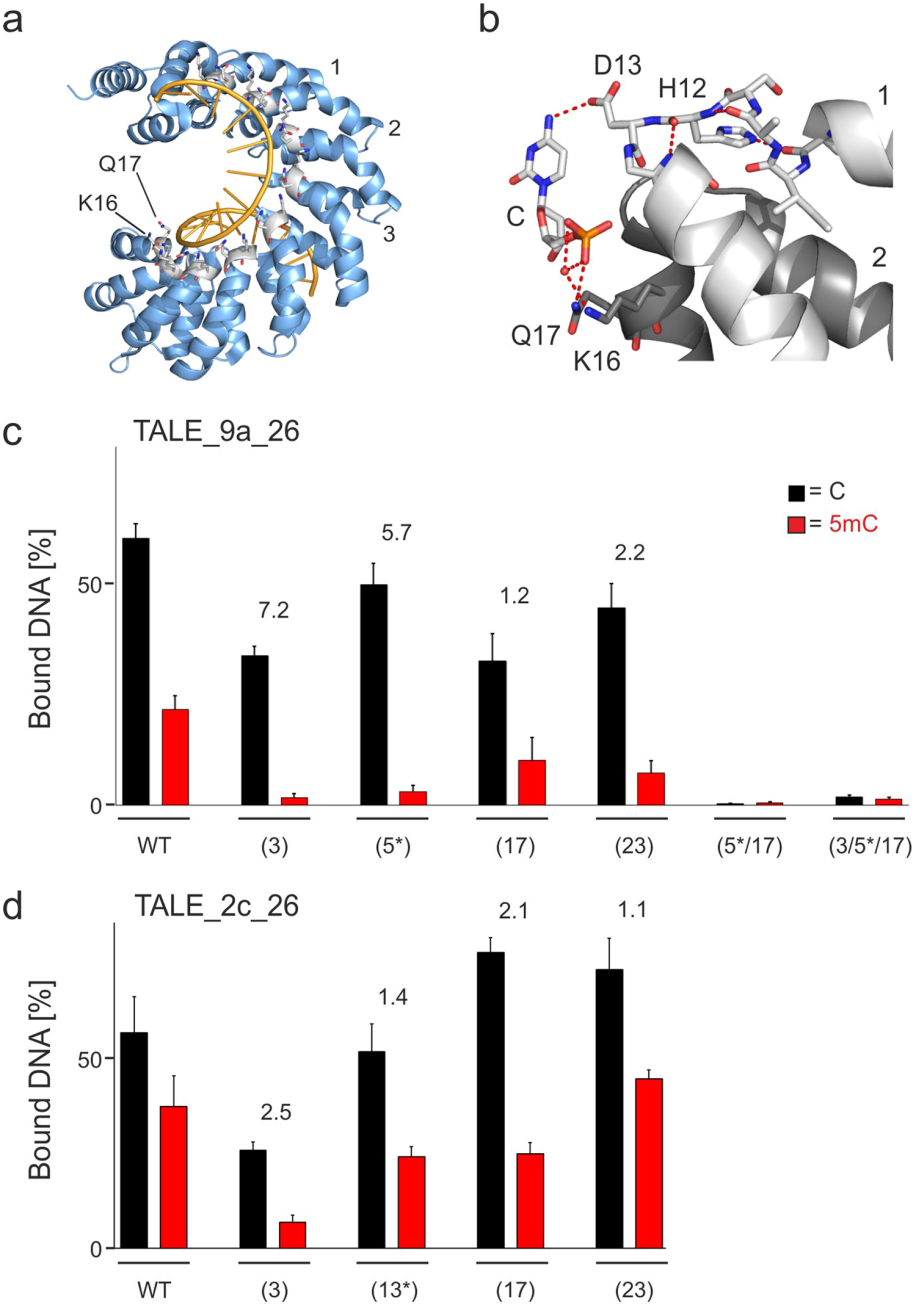
Influence of basic residues in the TALE CRD on affinity and C/5mC-selectivity. (**a**) Crystal structure of a TALE in DNA-bound state (pdb entry 3V6T)^33^. TALE is shown as blue cartoon, one strand of DNA is shown as orange cartoon, and DNA phosphate-binding residues K16 and Q17 of all regular CRD repeats are shown as sticks. (**b**) Interaction of RVD HD (amino acids 12 and 13 of TALE repeat) and residues K16 and Q17 with a C nucleobase in a crystal structure of a TALE-DNA complex (pdb entry 3V6T)^33^. Hydrogen bonds are shown as dotted red lines, water molecules are shown as red spheres. (**c**) Quantification of triplicate EMSA assays conducted as in Fig. 2e and f, with TALE_9a_26 and K16A/Q17A double mutants at CRD repeat positions as shown (*marks the repeat opposite the C/5mC-variable position). Fold change of selectivity over that of WT TALE_9a_26 is indicated for each TALE above columns. (**d**) Quantification of triplicate EMSA assays with TALE_2c_26 and K16A/Q17A double mutants at CRD repeat positions as shown. Fold change of selectivity over that of WT TALE_2c_26 is indicated for each TALE above columns.

We were next interested in evaluating basic residues in the regular TALE repeats of the CRD. Each regular repeat bears a GG diresidue at amino acid positions 14 and 15 adjacent to the RVD that undergoes water-mediated DNA phosphate interactions via its backbone amide groups^33,34^. Moreover, all CRD repeats contain a KQ diresidue at amino acid positions 16 and 17 (Fig. 3a shows overview of positions) that both interact with the 5′-phosphate of the preceeding nucleotide via their side chains, either direct (Q17) or water-mediated (K16) (Fig. 3b shows the interactions between repeat 2 and the 5′-phosphate of the C nucleotide opposite the preceeding repeat 1)^33,34^. Since the phosphate interactions of the GG diresidues are occuring via the backbone and their altering would require changes in the secondary structure of the TALE repeat loop (with unpredictable effects on the structure of the whole TALE repeat and TALE protein), we did not engineer this interaction. In contrast, K16A/Q17A double mutations should remove phosphate interactions with comparably low potential of disturbing the secondary structure of the TALE repeat^42^.

On the basis of the above TALE_9a_26, we constructed TALEs with K16A/Q17A double mutations in one to three CRD repeats. The positions of the mutated repeats were distributed over the CRD opposite different nucleobases, including the C/5mC-variable 5 position (marked as “5*”; other positions were 3, 17 and 23, Fig. 3c). Compared to the WT TALE_9a_26, all TALEs with K16A/Q17A double mutations in single repeats exhibited higher selectivity. The positions 3 and 5* in the CRD region close to the N-terminus thereby exhibited strong selectivity-increases of 5.7-fold and 7.2-fold compared to WT TALE_9a_26, whereas the repeats 17 and 23 close to the C-terminus showed only 1.2–2.2-fold increases (Fig. 3c). This potentially reflects a previously observed polarity in DNA binding of the TALE scaffold^50^ and several models which suggest that TALEs associate with DNA via 5′-repeats first, followed by 3′-repeats^40,51^. However, introduction of K16A/Q17A mutations into two (5* and 17) or three (3, 5* and 17) repeat positions resulted in abolishment of DNA binding (Fig. 3c).

We again employed TALE_2c_26 to perform an analogous experiment with K16A/Q17A mutations at repeat positions 3, 13* (opposite the variable C/5mC position), 17 and 23. Because of expected inactivity, we did not test mutations in two or more repeats. Though less pronounced as with TALE_9a_26, all mutants exhibited higher (1.1–2.5-fold) C/5mC selectivity than the WT TALE, with the highest selectivity for repeat 3 close to the N-terminus and the lowest selectivity for repeat 23 close to the C-terminus. These data show that removal of DNA phosphate-interacting amino acid side chains also in the CRD can result in TALE scaffolds with increased C/5mC selectivity. Compared to scaffolds with mutations in the NTR, CRD mutations can lead to overall higher selectivity, and mutation of repeats in the C-terminal CRD region result in lower selectivity increases than repeats in the N-terminal CRD region. Again, effects for the TALE_2c_26 sequence context and its more C-terminal C/5mC-variable position resulted in lower effects than observed for the TALE_9a_26 sequence context. Interestingly, K16A/Q17A mutations in repeats both opposite C nucleobases and G nucleobases resulted in TALEs with similar affinity to nonmethylated DNA as compared to the WT TALEs in several cases (Fig. 3c,d). This was observed for repeats 5* and 23 in TALE_9a_26 and repeats 13* and 23 in TALE_2c_26 (all positioned opposite C nucleobases) as well as repeat 17 in TALE_2c_26 (positioned opposite a G nucleobase; other positions opposite G nucleobases showed somewhat reduced binding, i.e. repeats 3 and 17 in TALE_9a_26 and repeat 3 in TALE_2c_26). This suggests that the K16A/Q17A double mutation does not strongly affect the structural preorganization and hydrogen bonding ability of HD RVD-containing repeats and potentially of NN RVD containing repeats, though a position dependence was observed for the latter.

### Combination of engineered N-terminal regions and central repeat domains

**Figure 4:**
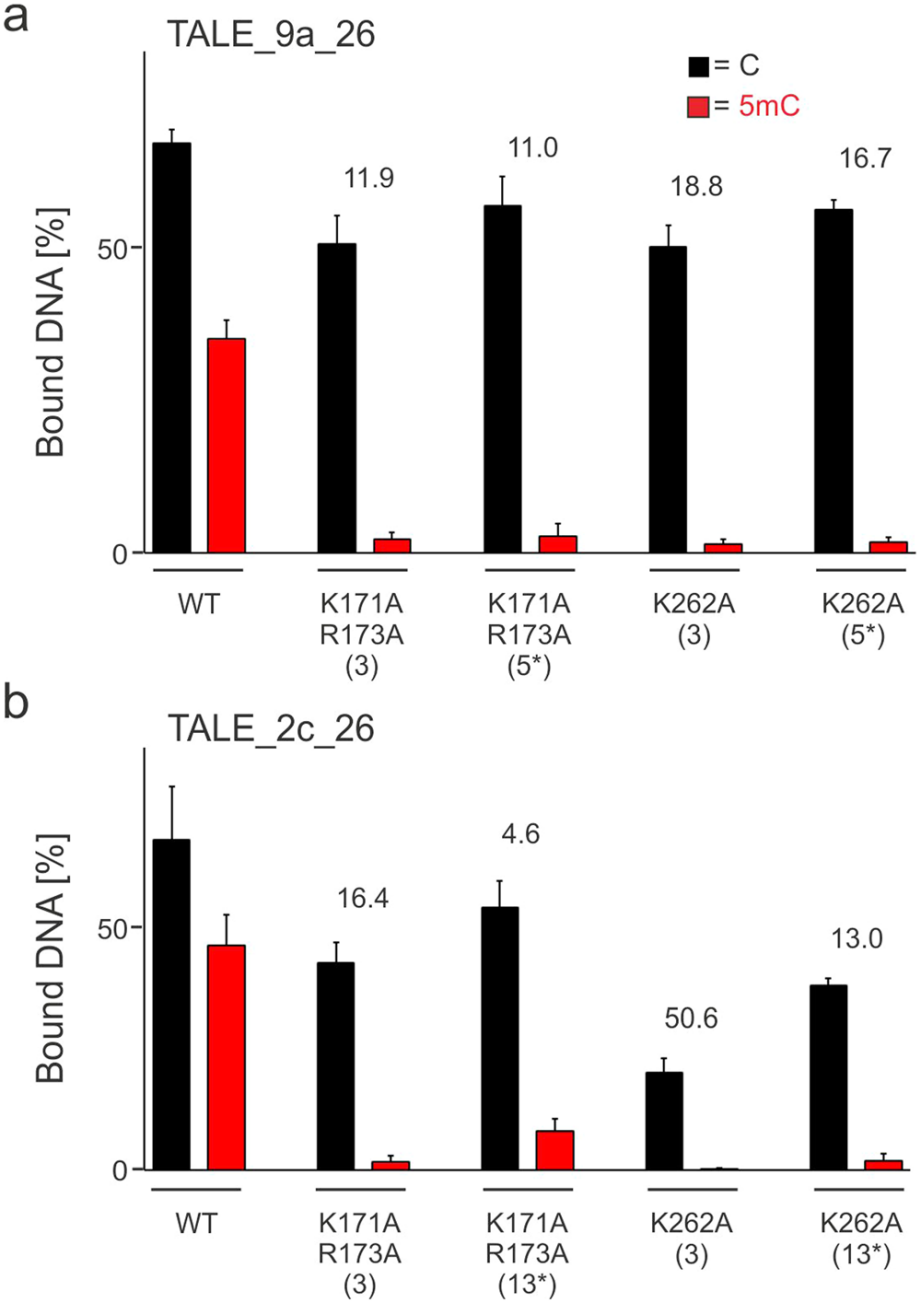
Combination of NTR and CRD alanine mutations to obtain TALE scaffolds with increased C/5mC selectivity of DNA binding. (**a**) Quantification of triplicate EMSA assays conducted as in Fig. 2e and f, with TALE_9a_26 containing both NTR alanine mutations and K16A/Q17A double mutations at CRD repeat positions as shown (*marks the repeat opposite the C/5mC variable position). Fold change of selectivity over that of WT TALE_9a_26 is indicated for each TALE above columns. (**b**) Quantification of triplicate EMSA assays with TALE_2c_26 containing both NTR alanine mutations and K16A/Q17A double mutations at CRD repeat positions as shown. Fold change of selectivity over that of WT TALE_2c_26 is indicated for each TALE above columns.

The observed enhancements in C/5mC-selectivity of TALEs with removed basic residues in either the NTR or CRD prompted us to test potential synergies in TALEs with combined mutations in both regions. For this, we constructed TALE_9a_26 mutants based on the two engineered NTR that previously exhibited the highest selectivities, K171/R173 and K262A (Fig. 2e,f). In these, we addionally introduced single engineered repeats in the CRD N-terminal region that we previously found to exhibit the highest selectivities, i.e. repeats 3 and 5* (Fig. 3c). Strikingly, compared to the WT TALE_9a_26, all constructs showed dramatically increased selectivity with little variation (11.0–18.8-fold, Fig. 4a). Moreover, when this engineering strategy was transferred to TALE_2c_26 (targeting repeats 3 and 13*) that exhibited overall lower selectivities than TALE_9a_26 in engineering experiments targeting only the NTR or CRD (Figs. 2f and 3d), a similarly dramatic increase in selectivity was observed, with slightly higher selectivity for TALE scaffolds containing the K262A NTR mutation (13.0–50.6-fold) compared to ones containing the K171A/R173A NTR double mutation (4.6–16.4-fold, Fig. 4b). These data show that - in contrast to the engineering of multiple CRD repeats that results in abolishment of DNA binding - engineering of a single NTR cryptic repeat combined with a single CRD repeat is synergetic and results in functional and highly C/5mC-selective TALE scaffolds. The finding that mutated CRD repeats at three different positions (3, 5*, 13*) resulted in a considerable selectivity enhancement indicates positional flexibility of the combination approach.

### Employment of engineered TALE scaffolds for affinity enrichment-based analysis of genomic 5mC with enhanced selectivity

**Figure 5:**
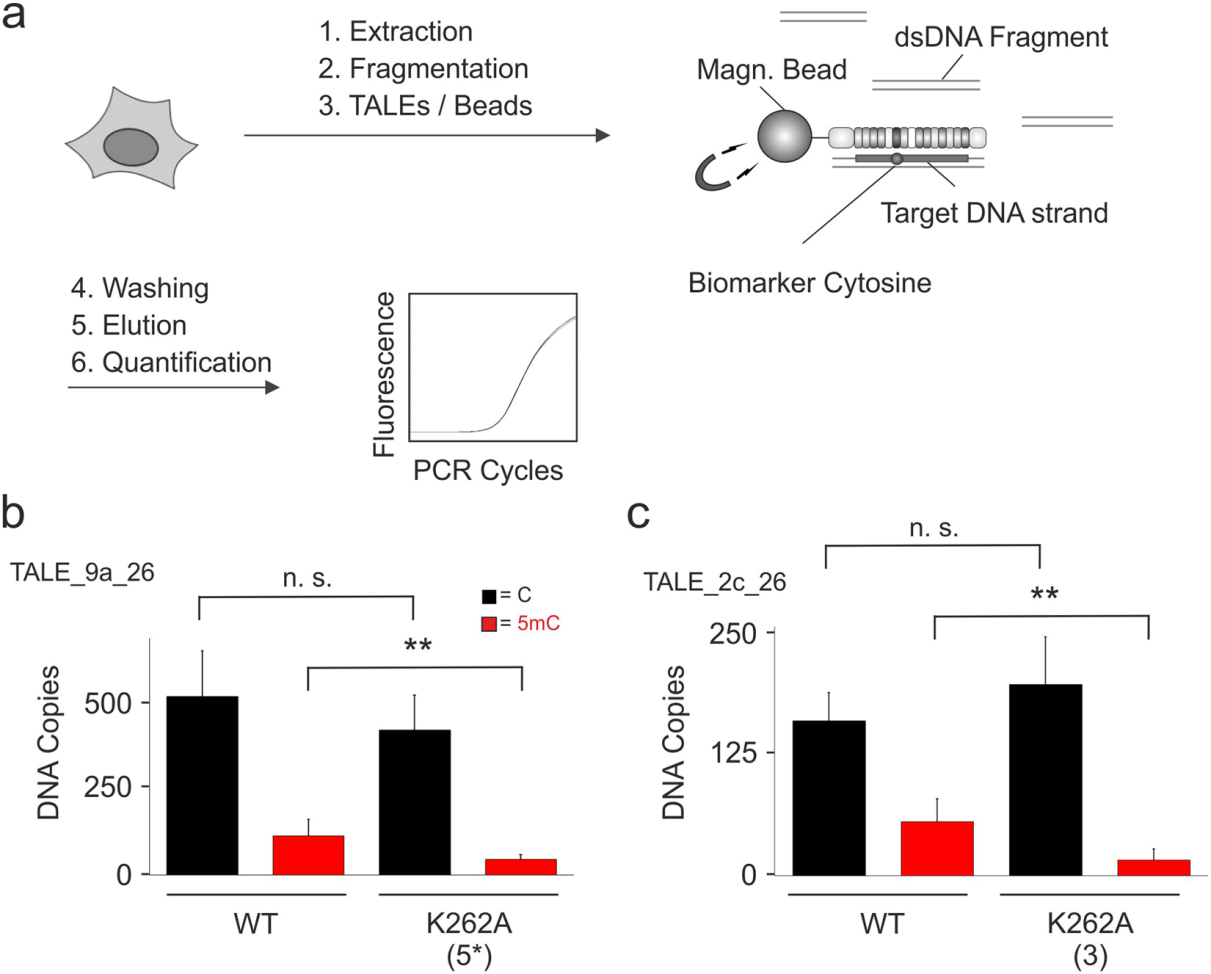
Engineered TALE scaffolds enable genomic 5mC detection with enhanced selectivity by affinity enrichment. (**a**) Workflow of TALE-based, 5mC-dependent affinity enrichment of user-defined genomic DNA sequences. (**b**) Affinity enrichment experiments with WT or engineered TALE_9a_26 as shown, using nonmethylated or methylated human genomic DNA. Target DNA copies obtained from enrichments were quantified by qPCR. Affinity enrichment experiments were conducted in triplicate and each quantified by duplicate qPCRs. **p < 0.01 from student t-test, n. s. = not significant. (**c**) Affinity enrichment experiments conducted as in Fig. 5b, with WT and engineered TALE_2c_26 as shown.

We have previously reported an assay for genomic 5mC detection based on solid phase affinity enrichment of DNA sequences with immobilized TALEs. This assay enables the detection of 5mC at single, user-defined genomic nucleotide positions in a strand-specific manner and exhibited a higher sensitivity than an antibody in the methylated DNA immunoprecipitation (MeDIP) protocol^39^. In this assay, TALEs are incubated with fragmented genomic DNA in solution and the formed TALE-DNA complexes are immobilized via a C-terminal His6 tag on the TALE protein to Ni-NTA magnetic agarose beads. After stringent washing, the bound DNA is eluted and quantified by qPCR (Fig. 5a).

To test, if the selectivity enhancements with the identified TALE mutants observed in EMSA were transferrable to this genomic assay with its drastically higher requirements of sensitivity and selectivity, we first tested TALE_9a_26 with an NTR K262A mutation and CRD repeat 5* K16A/Q17A mutations (Fig. 4a). Using 200 ng methylated or non-methylated input gDNA, we recovered similar copy numbers of nonmethylated target sequence for both TALEs, whereas less methylated target sequence copies were recovered with the mutant TALE, resulting in a 2-fold higher C/5mC selectivity (Fig. 5b). Moreover, when we performed the experiment with TALE_2c_26 bearing a K262A mutation in the NTR and K16A/Q17A mutations in CRD repeat 3 (Fig. 4b), we found a 4.2-fold higher selectivity for the mutant compared to the WT TALE, again with similar recovery of nonmethylated target sequence copies (Fig. 5c). These data show that the identified mutations indeed translate into enhanced C/5mC selectivities in affinity enrichment of target sequences from the human genome by a reduction of enrichment efficiency for 5mC containing DNA. Differences in selectivity increases between EMSA and genomic affinity enrichments observed for the engineered TALE scaffolds presumably originate from the far higher sequence complexity and lower target sequence concentration in the latter case. We cannot exclude that mutations in the engineered TALE scaffolds may e.g. affect the search kinetics of TALEs so that additional optimization of the here applied standard incubation/enrichment conditions are required to achieve further increased selectivities that better resemble the ones observed in EMSA assays.

### 5mC-selective transcription activation in human cells with engineered TALE scaffold

**Figure 6:**
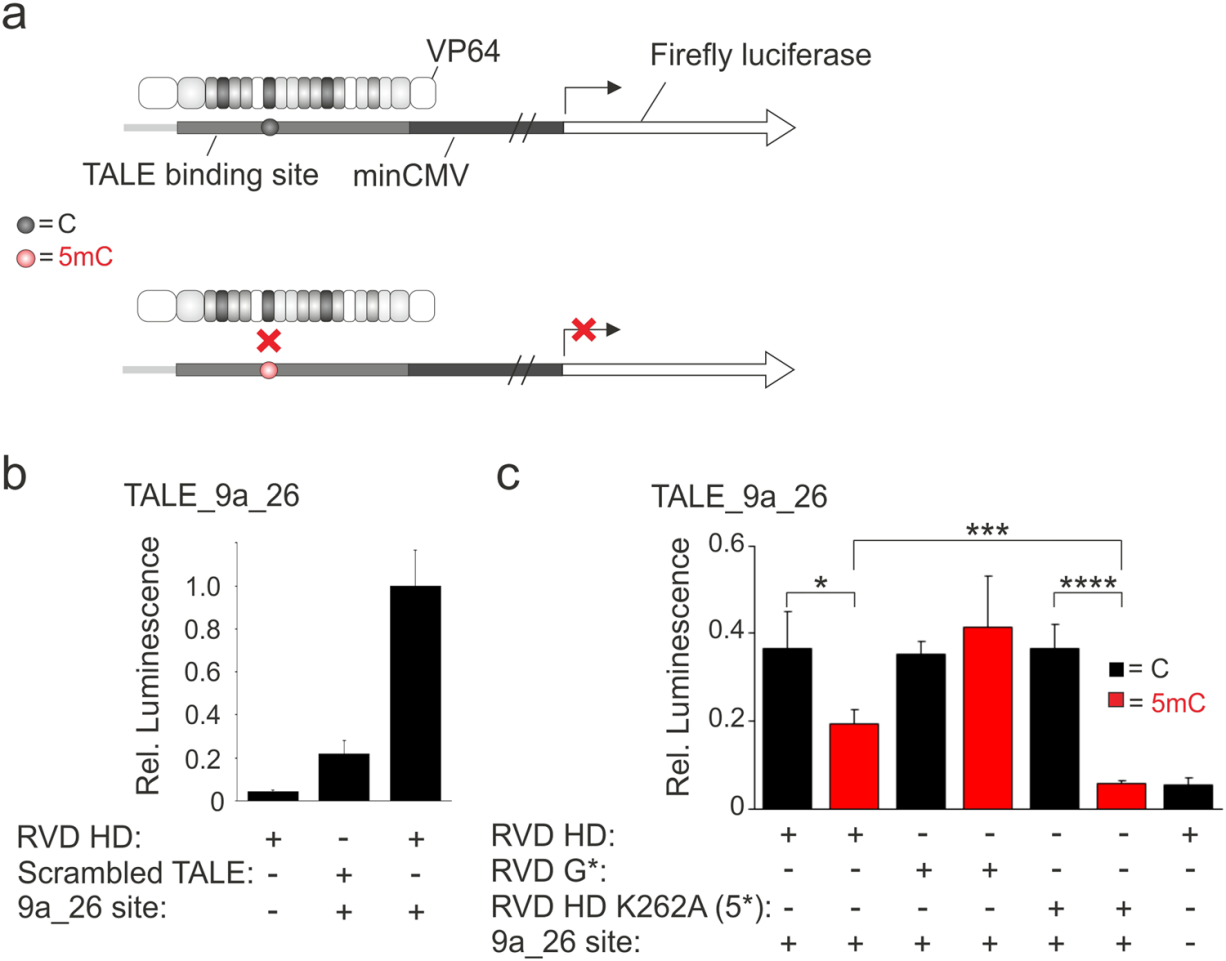
*In vivo* 5mC-selective transcriptional activation with engineered TALE scaffolds. (**a**) Principle of 5mC-selective luciferase reporter assay based on TALE-VP64 fusion construct. (**b**) Basic controls of transcriptional activation assay with different TALE and reporter gene constructs quantified by luminescence. Scrambled TALE is derived from TALE_9a_26 with same repeat composition but randomly chosen sequence, 9a_26 site is the binding site of TALE_9a_26 in the reporter as shown in Fig. 6a. (**c**) 5mC-dependent transcriptional activation of wild type TALE scaffolds with RVD HD and universal RVD G* or of engineered TALE scaffold with NTR and CRD alanine mutations as shown. Variable repeat is placed opposite 5 position in sequence 9a_26 (Fig. 2b), C or 5mC containing 9a_26 binding sites were introduced into reporter plasmid by ligation of synthetic DNA oligonucleotide duplexes and direct transfection. *p < 0.05, **p < 0.01 *** p < 0.005, ****p < 0.001 from student t-test of triplicate experiments.

TALEs are widely used scaffolds for *in vivo* genome targeting approaches, such as transcriptional activation/repression, genome engineering^24,25^, imaging of chromatin dynamics^52^ and the editing of epigenetic chromatin marks^53,54^. Achieving a high selectivity of these applications for defined 5mC positions in the TALE target sequences enables conditional control with perspectives for e.g. cell-type selective applications. A certain 5mC selectivity of RVD HD has previously been observed in *in vivo* genome engineering and transcriptional control^55,56^. To evaluate the potential of our new scaffold design for enhancement of selectivity for single 5mC positions in TALE target sequences *in vivo*, we tested a TALE_9a_26-based transcription factor in reporter gene activation assays. We first co-transfected an expression plasmid of WT TALE_9a_26 fused to a VP64 transcriptional activation domain with a reporter plasmid bearing the 9a_26 target sequence directly upstream of a minCMV promoter controlling expression of the firefly luciferase gene into HEK293T cells (Fig. 6a; for plasmid maps, see SI Fig. 5). Quantification of luciferase activity showed that transcription activation was dependent on selective binding of the TALE_9a_26 to its target sequence, as controls with a scrambled TALE with identical repeat composition as TALE 9a_26 but randomly altered repeat sequence and with a luciferase reporter not containing the 9a_26 sequence exhibited strongly reduced luciferase expression (Fig. 6b). To test for 5mC selectivity of the WT and engineered TALE scaffold, we ligated a synthetic oligonucleotide duplex containing or not containing a single 5mC nucleobase at position 5 of the TALE-bound strand in the 9a_26 sequence with a restricted luciferase reporter plasmid and directly transfected the purified ligation reactions together with the TALE-VP64 expression plasmids (bisulfite sequencing of re-isolated plasmids revealed stability of the 5mC nucleobase over the course of the luciferase expression experiment, see SI Fig. 6). Quantification of luciferase activity revealed a weak (1.8-fold) 5mC selectivity of WT TALE_9a_26 (Fig. 6c). This selectivity was due to C-selective binding of RVD HD, as a control TALE bearing the universal RVD G*^38,39^ at the same position did not exhibit 5mC-selectivity (Fig. 6c). However, TALE_9a_26 K262A (5*) exhibited the same transcriptional activation as the WT TALE for the non-methylated sequence, but a strongly reduced activation for the sequence containing a single 5mC, resulting in a 6.3-fold selectivity (Fig. 6c). Notably, luciferase activation for the 5mC sequence was reduced to the one of a background control, i.e. transfection of a luciferase plasmid not containing the 9a_26 sequence (subtraction of this background results in a theoretical 102-fold 5mC-selectivity of TALE_9a_26 K262A (5*)). These data show that the enhanced 5mC selectivity of our scaffold design is transferrable to TALE-based *in vivo* transcriptional activation.

## Conclusion

In summary, we employed mutational analysis to evaluate a larger set of basic residues throughout the TALE scaffold for their contribution to DNA binding affinity and C/5mC selectivity. Alanine mutagenesis of single to up to two residues within the NTR repeats partially resulted in moderate increases of C/5mC selectivity in EMSA studies, with K262A, R266A and K171A/R173A mutations exhibiting the most pronounced effects. In contrast, alanine mutation of more than two residues impaired DNA binding. When introducing K16A/Q17A double mutations in the CRD repeats, stronger selectivity enhancements were observed in several cases, and mutations in the N-terminal CRD region seemed to have higher impact than ones in the C-terminal region, potentially reflecting a previously observed polarity of the TALE scaffold^50^ and previously suggested binding mechanisms of TALEs^40,51^. Mutation of two or more CRD repeats impaired DNA binding. However, the strongest selectivity increases were observed for combined mutations in the NTR and CRD, which resulted in ≥11-fold enhanced C/5mC selectivity compared to the WT TALE in seven of eight cases. In general, increased selectivity was a result of reduced binding to 5mC-containing DNA rather than an increase in binding to C-containing DNA. This suggests that electrostatic interactions are more dominant in binding to 5mC-containing DNA as compared to C-containing DNA and argues for the formation of alternative TALE-DNA complex types in the two cases. Employment of two of the new TALE scaffolds in solid-phase affinity enrichment of DNA sequences revealed up to 4.2-fold enhanced C/5mC selectivity also in the context of the human genome. Moreover, a 3.5-fold increased single 5mC-selectivity was observed in *in vivo* transcriptional activation with a TALE-VP64 construct based on the altered scaffold design. These TALE scaffolds will be useful for the selective analysis of 5mC at single, user-defined genomic nucleotide positions and provide improved perspectives for the 5mC-conditional control of TALE-based *in vivo* applications.

## Electronic supplementary material

SI
