# Supplementary material for: Engineering DNA Backbone Interactions Results in TALE Scaffolds with Enhanced 5-Methylcytosine Selectivity
Source: Sci Rep. 2017 Nov 8;7:15067. doi: 10.1038/s41598-017-15361-1 (PMC5678105; doi:10.1038/s41598-017-15361-1)

Supporting Online Information

Engineering DNA Backbone Interactions Results in TALE Scaffolds with Enhanced 5-Methylcytosine Selectivity

Preeti Rathi, Anna Witte and Daniel Summerer*

Department of Chemistry and Chemical Biology

Technical University of Dortmund

CP-02-143

Otto-Hahn-Str. 4a

44227 Dortmund

*Daniel.Summerer@tu-dortmund.de

Table of Contents

List of Oligonucleotides 3

Figure SI 1: Protein Sequences of Exemplary TALE proteins with N-terminal GFP domain and C- terminal His-tag 5

Figure SI 2: SDS PAGE Analysis of Exemplary Ni-NTA-Purified TALE Protein Expression (TALE_9a_26 and TALE_2c_26) 6

Figure SI 3: Agarose gel analysis of human whole genome amplified DNA randomly sheared by sonication 7

Figure SI 4: Sanger Sequence traces of PCR products from bisulfite converted human DNA before and after enzymatic CpG methylation 8

Figure SI 5: Map of plasmids used in TALE-based in vivo transcription activation 9

Figure SI 6: Stability of synthetically introduced 5mC nucleotide during in vivo transcription activation assay shown in Figure 6c 10

Figure SI 7: Influence of number of amino acids changed on TALE binding and selectivity 11

Figure SI 8: Influence of number of methylated CpG on TALE binding and selectivity 12

Figure SI 9: Genomic sequences around TALE targets.................................. 13

Figure SI 10: Original EMSA gels from Figure 2d……………………………….14

List of Oligonucleotides

All Oligos were ordered from Sigma-Aldrich. 5-methyl-C nucleotides are marked as **C**.

Quantitative real time PCR

9a_26_Fw (oPrR2054) GTC GGG TAG AGG AGG TGC G

9a_26_Rv (oPrR2053) GGC TCC TCA TTC CTC TTC CTT G

2c_26_Fw (oPrR1759) GCT CTG GGA GAC CTG

2c_26_Rv (oPrR1764) CGG TTC TGG GTG AGG

Electromobility shift assay (EMSA)

9a_26_C_fw (oGrK1591) GGC CAG CCA GTC AGC CGA AGG CTC CAT GCT GCT CCC CGC CGC CGG C

9a_26_mC_fw (oGrK1592) GGC CAG CCA GTC AGC **C**GA AGG CTC CAT GCT GCT CCC CGC CGC CGG C

9a_26 _rv (oGrK1617) GCC GGC GGC GGG GAG CAG CAT GGA GCC TTC GGC TGA CTG GCT GGC C

2c_26_C_fw (oPrR2121) CAT CCA TGA GTG AGA AAC CCT GGC GGG GTG TGA CAT CCT CCC CCG G

2c_26_mC_fw (oPrR2123) CAT CCA TGA GTG AGA AAC CCT GG**C** GGG GTG TGA CAT CCT CCC CCG G

2c_26 _rv (oPrR2122) CCG GGG GAG GAT GTC ACA CCC CGC CAG GGT TTC TCA CTC ATG GAT G

TALE cloning

pAnI521_K171A_fw (oPrR1918) GCA ACA GGA GAA AAT CAA GCC TGC CGT CAG GAG CAC CGT CGC GCA AC

pAnI521_K171A_rv (oPrR1919) GTG TTG CGC GAC GGT GCT CCT GAC GGC AGG CTT GAT TTT CTC CTG TTG

pAnI521_R173A_fw (oPrR1920) GGA GAA AAT CAA GCC TAA GGT CGC CAG CAC CGT CGC GCA ACA CCA C

pAnI521_R173A_rv (oPrR1921) CGT GGT GTT GCG CGA CGG TGC TGG CGA CCT TAG GCT TGA TTT TCT C

pAnI521_R236A_fw (oPrR1922) GTA AAC AGT GGT CGG GAG CGG CCG CAC TTG AGG CGC TGC TGA CTG

pAnI521_R236A_rv (oPrR1923) GTC AGC AGC GCC TCA AGT GCG GCC GCT CCC GAC CAC TGT TTA CC
pAnI521_R266A_fw (oPrR1924) CAG CTG CTG AAG ATC GCG AAG GCC GGG GGA GTA ACA GCG GTA G

pAnI521_R266A_rv (oPrR1925) CTC TAC CGC TGT TAC TCC CCC GGC CTT CGC GAT CTT CAG CAG C
pAnI521_K171A&R173A_fw (oPrR1926) AGG AGA AAA TCA AGC CTG CCG TCG CCA GCA CCG TCG CGC AAC ACC A

pAnI521_ K171A&R173A _rv (oPrR1927) GTG TTG CGC GAC GGT GCT GGC GAC GGC AGG CTT GAT TTT CTC CTG TTG

pAnI521_K262A_fw (oPrR1928) CAC CGG GCA GCT GCT GGC CAT CGC GAA GAG AGG GGG AG

pAnI521_ K262A_rv (oPrR1929) CCC CTC TCT TCG CGA TGG CCA GCA GCT GCC CGG TGT CG

pPrR771_K265A&R266A_fw (oPrR1930) CAG CTG CTG GCC ATC GCG GCC GCC GGG GGA GTA ACA GCG GT

pPrR771_K265A&R266A_rv (oPrR1931) CGC TGT TAC TCC CCC GGC GGC CGC GAT GGC CAG CAG CTG C

pNN3_K16A&Q17A_fw (oPrR1932) CGC CAG CAA CAA TGG CGG CGC CGC CGC GCT CGA AAC GGT GCA GCG

pNN3_K16A&Q17A_rv (oPrR1933) GCT GCA CCG TTT CGA GCG CGG CGG CGC CGC CAT TGT TGC TGG CGA TAG

pNN7_K16A&Q17A_fw (oPrR1934) CGC CAG CAA CAA TGG CGG CGC CGC CGC GCT CGA AAC GAG ACC CTC

pNN7_K16A&Q17A_rv (oPrR1935) GGG TCT CGT TTC GAG CGC GGC GGC GCC GCC ATT GTT GCT GGC GAT AG

pHD3_K16A&Q17A_fw (oPrR1936) CGC CAG CCA CGA TGG CGG CGC CGC CGC GCT CGA AAC GGT GCA GC

pHD3_K16A&Q17A_rv (oPrR1937) CTG CAC CGT TTC GAG CGC GGC GGC GCC GCC ATC GTG GCT GGC GAT AG

pHD5_K16A&Q17A_fw (oPrR1938) CGC CAG CCA CGA TGG CGG CGC CGC CGC GCT CGA AAC GGT GCA GG

pHD5_K16A&Q17A_rv (oPrR1939) CTG CAC CGT TTC GAG CGC GGC GGC GCC GCC ATC GTG GCT GGC GAT AG

TALE-based in vivo transcription activation with mC-selectivity

9a_26_C_fw (oAnW2016) TTT TGT CGA CTC AGC CGA AGG CTC CAT GCT GCT CCC ACT AGT TTT T

9a_26_mC_fw (oPrR2248) TTT TGT CGA CTC AGC **C**GA AGG CTC CAT GCT GCT CCC ACT AGT TTT T

9a_26_rv (oAnW2017) AAA AAC TAG TGG GAG CAG CAT GGA GCC TTC GGC TGA GTC GAC AAA A

Bisulphite PCR / Sequencing

Fw1 (oPrR1307) GAA ATT GGA GAT TTT TAT TAG GG

Rv1 (oPrR1308) TAT CTA AAA AAC CCC ACA ACC TAT C

Fw2 (oPrR1256) TGG GGG ATT GGG ATT TTT TTT

Rv2 (oPrR1255) AAT AAA AAA AAC AAA AAA AAC CAA AC

Rv3 (oPrR1257) TTA ACC ACC CAA TCT ACC CCC

CDKN2A_Fw1 (oPrR2425) TAG TGG TTA TAA TAG TAG TTT TAG TT

CDKN2A _Rv1 (oPrR2426) ATA ATT TAT ATT CAA CCC ATA A

CDKN2A _Fw2 (oPrR2427) GAG GTT TTA AGG GGT TAT GTT ATT AAT

CDKN2A _Rv2 (oPrR2424) AAC ATC TTC CAT AAT AAA TAC CTC CT

Figure SI 1: Protein Sequences of Exemplary TALE proteins with N-terminal GFP domain and C- terminal His-tag

GFP_TALE_9a_26

MSKGEELFTGVVPILVELDGDVNGHKFSVSGEGEGDATYGKLTLKFICTTGKLPVPWPTLVTTLTYGVQCFSRYPDHMKQ
HDFFKSAMPEGYVQERTIFFKDDGNYKTRAEVKFEGDTLVNRIELKGIDFKEDGNILGHKLEYNYNSHNVYIMADKQKNG
IKANFKIRHNIEDGSVQLADHYQQNTPIGDGPVLLPDNHYLSTQSALSKDPNEKRDHMVLLEFVTAAGITLGMDELYKTL
GYSQQQQEKIKPKVRSTVAQHHEALVGHGFTHAHIVALSQHPAALGTVAVKYQDMIAALPEATHEAIVGVGKQWSGARAL
EALLTVAGELRGPPLQLDTGQLLKIAKRGGVTAVEAVHAWRNALTGAPLNLTPDQVVAIASHDGGKQALETVQRLLPVLC
QDHGLTPDQVVAIASNIGGKQALETVQRLLPVLCQDHGLTPDQVVAIASNNGGKQALETVQRLLPVLCQDHGLTPDQVVA
IASHDGGKQALETVQRLLPVLCQDHGLTPDQVVAIASHDGGKQALETVQRLLPVLCQDHGLTPDQVVAIASNNGGKQALE
TVQRLLPVLCQDHGLTPDQVVAIASNIGGKQALETVQRLLPVLCQDHGLTPDQVVAIASNIGGKQALETVQRLLPVLCQD
HGLTPDQVVAIASNIGGKQALETVQRLLPVLCQDHGLTPDQVVAIASNNGGKQALETVQRLLPVLCQDHGLTPDQVVAIA
SHDGGKQALETVQRLLPVLCQDHGLTPDQVVAIASNGGGKQALETVQRLLPVLCQDHGLTPDQVVAIASHDGGKQALETV
QRLLPVLCQDHGLTPDQVVAIASHDGGKQALETVQRLLPVLCQDHGLTPDQVVAIASNIGGKQALETVQRLLPVLCQDHG
LTPDQVVAIASNGGGKQALETVQRLLPVLCQDHGLTPDQVVAIASNNGGKQALETVQRLLPVLCQDHGLTPDQVVAIASH
DGGKQALETVQRLLPVLCQDHGLTPDQVVAIASNGGGKQALETVQRLLPVLCQDHGLTPDQVVAIASNNGGKQALETVQR
LLPVLCQDHGLTPDQVVAIASHDGGKQALETVQRLLPVLCQDHGLTPDQVVAIASNGGGKQALETVQRLLPVLCQDHGLT
PDQVVAIASHDGGKQALETVQRLLPVLCQDHGLTPDQVVAIASHDGGKQALETVQRLLPVLCQDHGLTPDQVVAIASHDG
GKQALESIVAQLSRPDPALAALTNDHLLEHHHHHH*

GFP_TALE_2c_26

MSKGEELFTGVVPILVELDGDVNGHKFSVSGEGEGDATYGKLTLKFICTTGKLPVPWPTLVTTLTYGVQCFSRYPDHMKQ
HDFFKSAMPEGYVQERTIFFKDDGNYKTRAEVKFEGDTLVNRIELKGIDFKEDGNILGHKLEYNYNSHNVYIMADKQKNG
IKANFKIRHNIEDGSVQLADHYQQNTPIGDGPVLLPDNHYLSTQSALSKDPNEKRDHMVLLEFVTAAGITLGMDELYKTL
GYSQQQQEKIKPKVRSTVAQHHEALVGHGFTHAHIVALSQHPAALGTVAVKYQDMIAALPEATHEAIVGVGKQWSGARAL
EALLTVAGELRGPPLQLDTGQLLKIAKRGGVTAVEAVHAWRNALTGAPLNLTPDQVVAIASNNGGKQALETVQRLLPVLC
QDHGLTPDQVVAIASNIGGKQALETVQRLLPVLCQDHGLTPDQVVAIASNNGGKQALETVQRLLPVLCQDHGLTPDQVVA
IASNIGGKQALETVQRLLPVLCQDHGLTPDQVVAIASNIGGKQALETVQRLLPVLCQDHGLTPDQVVAIASNIGGKQALE
TVQRLLPVLCQDHGLTPDQVVAIASHDGGKQALETVQRLLPVLCQDHGLTPDQVVAIASHDGGKQALETVQRLLPVLCQD
HGLTPDQVVAIASHDGGKQALETVQRLLPVLCQDHGLTPDQVVAIASNGGGKQALETVQRLLPVLCQDHGLTPDQVVAIA
SNNGGKQALETVQRLLPVLCQDHGLTPDQVVAIASNNGGKQALETVQRLLPVLCQDHGLTPDQVVAIASHDGGKQALETV
QRLLPVLCQDHGLTPDQVVAIASNNGGKQALETVQRLLPVLCQDHGLTPDQVVAIASNNGGKQALETVQRLLPVLCQDHG
LTPDQVVAIASNNGGKQALETVQRLLPVLCQDHGLTPDQVVAIASNNGGKQALETVQRLLPVLCQDHGLTPDQVVAIASN
GGGKQALETVQRLLPVLCQDHGLTPDQVVAIASNNGGKQALETVQRLLPVLCQDHGLTPDQVVAIASNGGGKQALETVQR
LLPVLCQDHGLTPDQVVAIASNNGGKQALETVQRLLPVLCQDHGLTPDQVVAIASNIGGKQALETVQRLLPVLCQDHGLT
PDQVVAIASHDGGKQALETVQRLLPVLCQDHGLTPDQVVAIASNIGGKQALETVQRLLPVLCQDHGLTPDQVVAIASNGG
GKQALESIVAQLSRPDPALAALTNDHLLEHHHHHH*

Color code:

K171, R173, R236, K262, K265, R266, K16A, Q17A

Figure SI 2: SDS PAGE Analysis of Exemplary Ni-NTA-Purified TALE Protein Expression (TALE_9a_26 and TALE_2c_26)

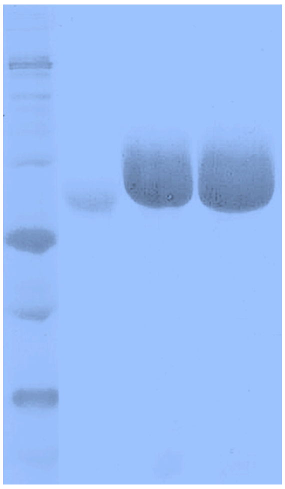

Lane 1: 6.25 - 212 kDa ladder (NEB)

Lane 2: non induced culture

Lane 3: purified first elution of expression culture transformed with pET_GFP_TALE_9a_26 induced with 1mM IPTG

Lane 4: purified first elution of expression culture transformed with pET_GFP_TALE_2c_26 induced with 1mM IPTG

Figure SI 3: Agarose gel analysis of human whole genome amplified DNA randomly sheared by sonication

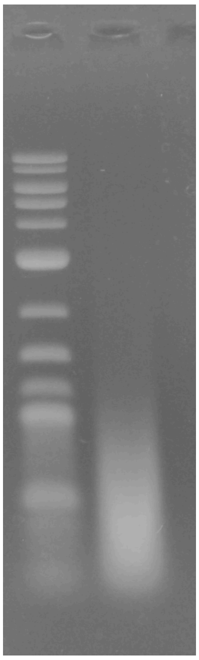

Lane 1: 2-log DNA ladder (New England Biolabs)

Lane 2: Fragmented whole genome amplified human DNA (100ng)

Figure SI 4: Sanger Sequence traces of PCR products from bisulfite converted human DNA before and after enzymatic CpG methylation

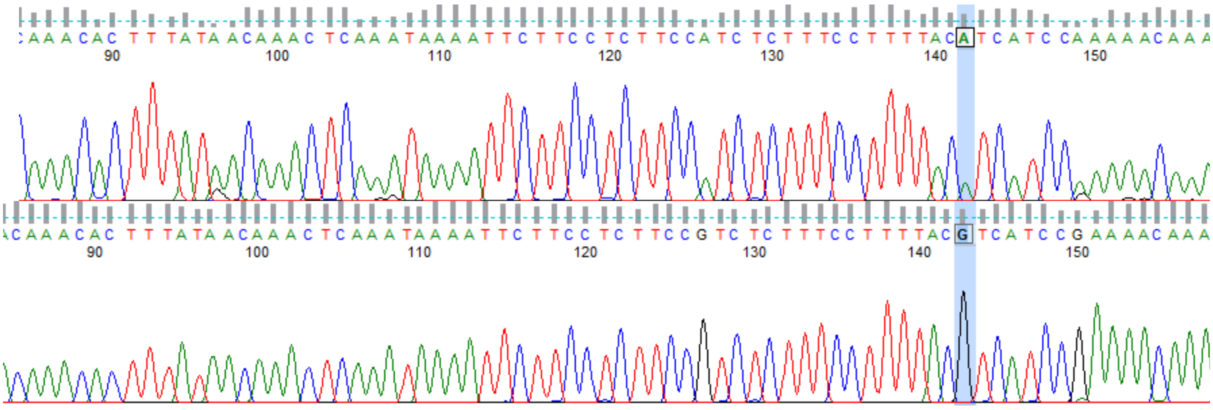

Upper Trace: original *H. sapiens* DNA

Lower Trace: Methylated *H. sapiens* DNA

Example CpG marked blue

Figure SI 5: Map of plasmids used in TALE-based in vivo transcription activation

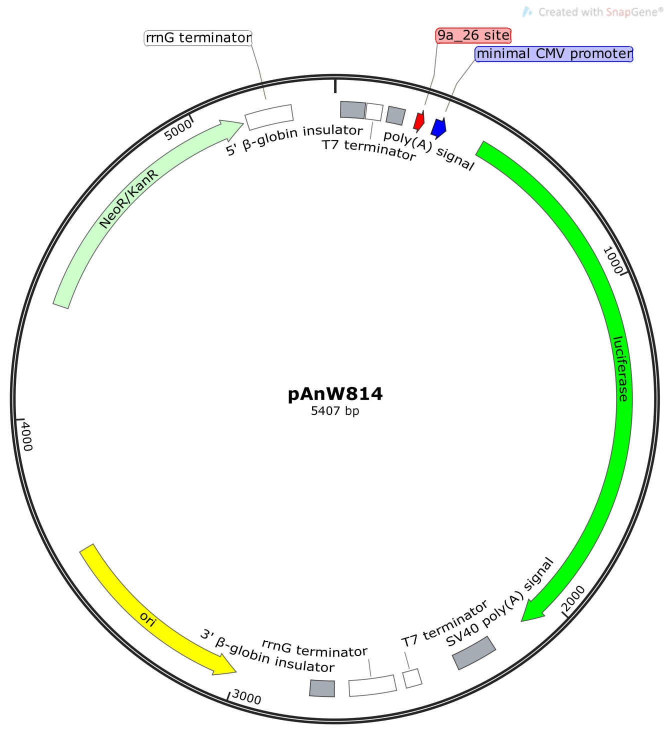

Plasmid group A carrying a TALE binding site, a minCMV promoter and the firefly luciferase gene.

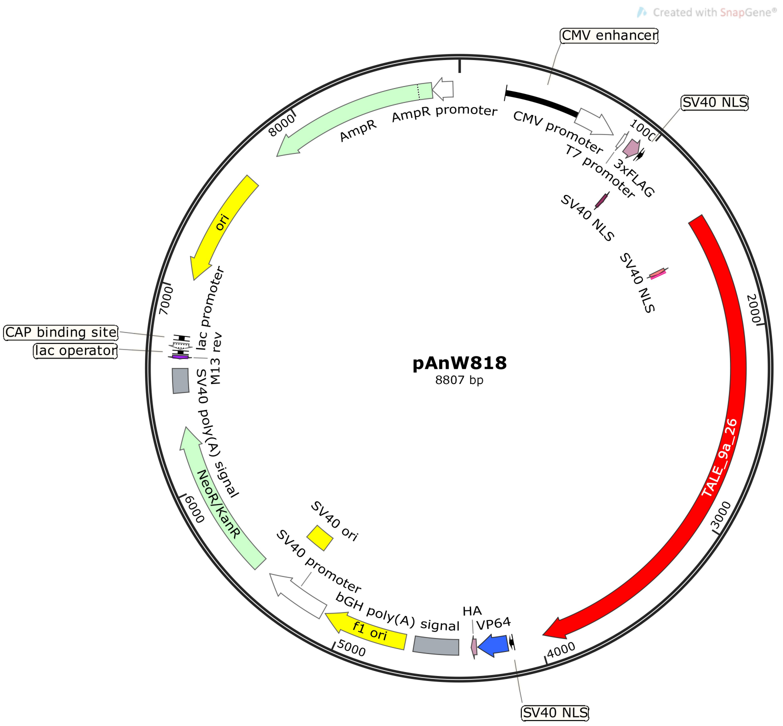

Plasmid group B carrying sequence for TALE and VP64 under the effect of CMV promoter

Figure SI 6: Stability of synthetically introduced 5mC nucleotide during in vivo transcription activation assay shown in Figure 6c

DNA from cells transfected with luciferase reporter plasmids obtained by ligation of synthetic DNA oligonucleotide duplexes bearing a single C or 5mC in the TALE_9a_26 binding site was isolated after identical treatment and cultivation as cells used for luciferase assay and bisulfite converted. TALE_9a_26 binding site was amplified as described and bisulfite sequenced.

DNA from cells transfected with C-containing plasmid

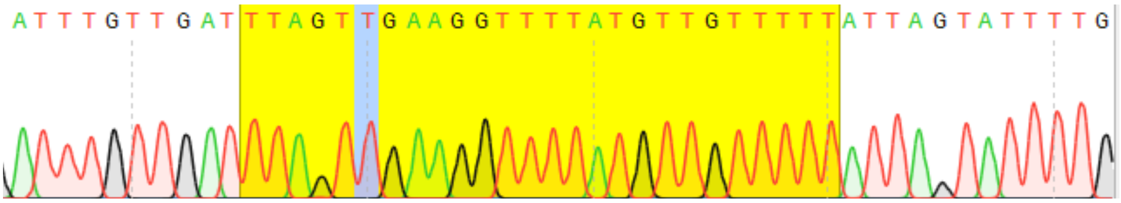

DNA from cells transfected with 5mC-containing plasmid

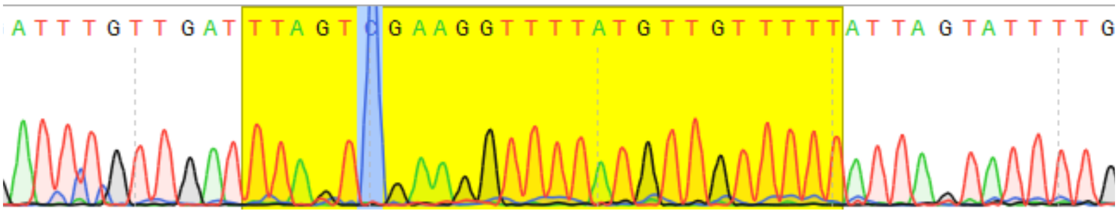

TALE_9a_26 binding site is marked yellow, variable C-position of CpG in blue.

Figure SI 7: Influence of number of amino acids changed on TALE binding and selectivity

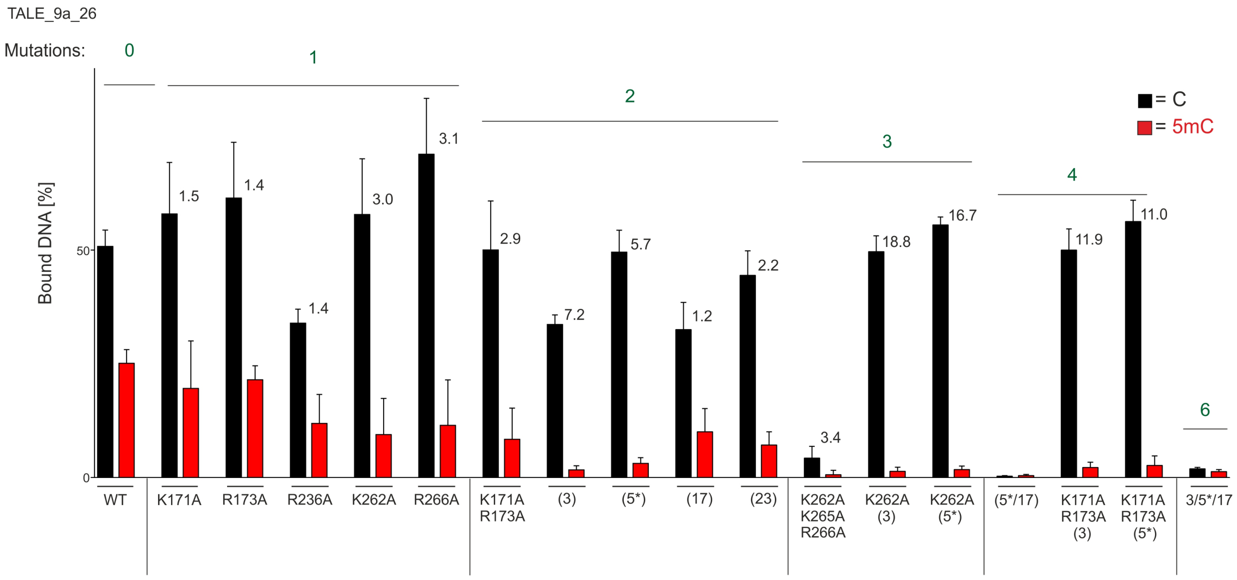

Result displaying quantification of triple EMSA assays with all TALE_9a_26 used in the study.

The overall relationship between the number of amino acids changed and improvement in C/5mC selectivity cannot be judged without including factors like the position of the mutation. The candidates that displayed the best selectivity have a single lysine in the NTR and two adjacent lysines and glutamines at positions 16 and 17 in a repeat targeting cytosine. However, when the changes are not distributed and all three of them occur in the NTR (K262A/K265A/R266A) there is extreme decrease in TALE binding. Similarly, four mutations (two each in the NTR and in the CRD) also enhanced TALE selectivity. However, when all the four mutations are present in the CRD, binding is severely affected. In addition to the number of amino acids changed, their position plays a critical role in deciding the effect on TALE function and sensitivity. In the NTR, amino acid positions K262 and K171/R173 seem to play a major role in TALE selectivity, whereas mutations in the CRD seem to follow the earlier observed effect of repeats closer to the NTR contributing more to TALE selectivity then those further away.

Figure SI 8: Influence of number of methylated CpG on TALE binding and selectivity

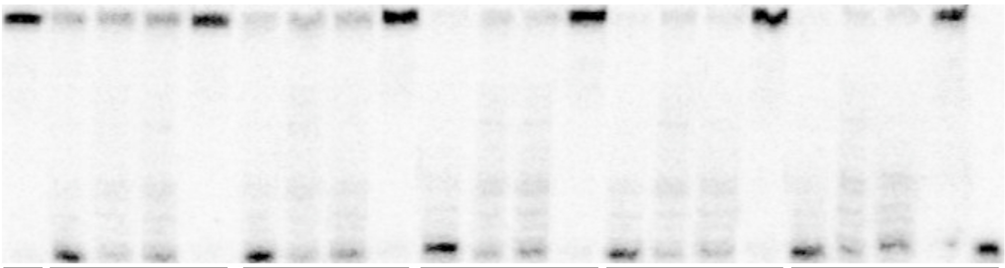

TALE/DNA	0	10				20				30				40				50				
TALE_BRCA1	-	+	+	+	+	+	+	+	+	+	+	+	+	+	+	+	+	+	+	+	+	+
C13	+	+	-	+	-	+	-	+	-	+	-	+	-	+	-	+	-	+	-	+	-	+
C20	+	+	+	-	-	+	+	-	-	+	+	-	-	+	+	-	-	+	+	-	-	+
mC13	-	-	+	-	+	-	+	-	+	-	+	-	+	-	+	-	+	-	+	-	+	-
mC20	-	-	-	+	+	-	-	+	+	-	-	+	+	-	-	+	+	-	-	+	+	-
KF	+	+	+	+	+	+	+	+	+	+	+	+	+	+	+	+	+	+	+	+	+	-

Primer extension assay performed as described previously (G. Kubik et. al. *Angew. Chem. Int. Ed.*, **2014**, 53, 6002-6.) using TALE BRCA1 (26) and synthetic oligonucleotide with varying number of methylated CpG using 250 mU of KF $^{(exo-)}$ per reaction.

BRCA1(26) TALE target sequence:

1 2 3 4 5 6 7 8 9 10 11 12 13 14 15 16 17 18 19 20 21 22 23 24 25 26

T C T T T C C T T T T A **C G** T C A T C **C G** G G G G C

The two CpGs denoted as C/5mC 13 and 20 in the figure are shown in bold and underlined

Figure SI 9: Genomic sequences around TALE targets

TALE_9a_26

CATTCCCATCTCTCAGTCCCATCCTGCAGCTGGGCAGGCAGTGCTGGGCCCCGGAAATGCCCTC
TGCCTCCCTGGAGCACGTGGCCTGTGATTTTCCTTGAGCACAGCACTTTGTGACTTTGATGTAAA
CATCAAACACAGCCCCCTTTCCTGTCTTCGCATCCAGGAAATAGGTTAGTTTCAGACAAGCCTGC
TTGCCGGAGCTCAGCAGACACCAGGCCTTCCGGGCAGGCCTGGCCCACCGTGGGCCTCAGAGC
TGCTGCTGGGGCATTCAGGTAAGCGGCTGTCCTCGGGCCTTCTGCCTGTGTCCTGCCACCAGG
GCCACCCCAGGGGGCTCTGGGAGACCTGGCAGAGGATGAACCCCCCCCATCCATGAGTGAGAA
ACCCTGGCGGGGTGTGACATCCTCCCCCGGGGTGTGTGCGGAGAGTGTGAGCGGCAGGGCCT
CCCTCTCAGGCTGGGGGCTTGGGCTGCAGGGAAATCCAAACCGGCTTTGTAAGCCCCGATTCCT
CACCCAGAACCGGCTCTCCATTGGCATTGGGACCAGAGACCCCGCAAGTGGCCTGTTTGCCTGG
ACATCCACCTGTACGTCCCCAGGTAAGTGGTGCCTGGGTGGAGTGGCACCTGGCCACCGTCTCC
ATGGTGGGCTGTGTCTGGAGGAGGAAGACAGAATATTAACTGGGAGTCAGGAGGTTGCTGGGA

TALE_2c_26

AATACAAATATGTTCCCCCCTTCAGATCTTCTCAGCATTCGAGAGATCTGTACGCGCGTGGCTCC
TCATTCCTCTTCCTTGGCTTCCCAAGCCCCCAGGGCGTCGCCAGGAGGAGGTCTGTGATTACAA
ACCCCTTCTGAAAACTCCCCAGGAAGCCTCCCCTTTTTCCGGAGAATCGAAGCGCTACCTGATTC
CAATTCCCCTGCAAACTTCGTCCTCCAGAGTCGCCCGCCATCCCCTGCTCCCGCTGCAGACCCT
CTACCCACCTGGATCGGCCTCCGACCGTAACTATTCGGTGCGTTGGGCAGCGCCCCCGCCTCCA
GCAGCGCCCGCACCTCCTCTACCCGACCCCGGGCCGCGGCCGTGGCCAGCCAGTCAGCCGAA
GGCTCCATGCTGCTCCCCGCCGCCGGCTCCATGCTGCTCCCCGCCGCCCGCTGCCTGCTCTCC
CCCTCTCCGCAGCCGCCGAGCGCACGCGGTCCGCCCCACCCTCTGGTGACCAGCCAGCCCCTC
CTCTTTCTTCCTCCGGTGCTGGCGGAAGAGCCCCCTCCGACCCTGTCCCTCAAATCCTCTGGAG
GGACCGCGGTATCTTTCCAGGCAAGGGGACGCCGTGAGCGAGTGCTCGGAGGAGGTGCTATTA
ACTCCGAGCACTTAGCGAATGTGGCACCCCTGAAGTCGCCCCAGGTTGGGTCTCCCCCGGGGG
CACCAGCCGGAAGCAGCCCTCGCCAGAGCCAGCGTTGGCAAGGAAGGAGGACTGGGCTCCTCC
CCACCTGCCCCCCACACCGCCCTCCGGCCTCCCTGCT

TALE target sequences are marked grey, CpG dinculeotides in red.

Figure SI 10: Original EMSA gels from Figure 2d

WT: Lanes 1, 2 and 11

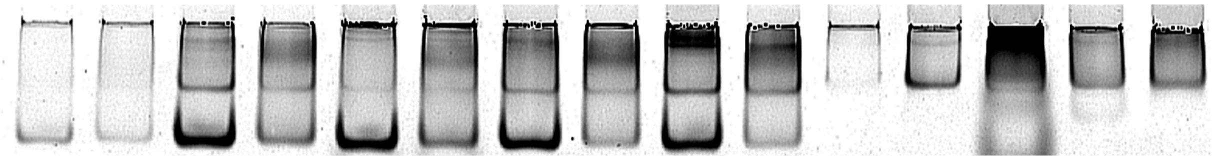

TALE_9a_26: Lanes 3 - 8

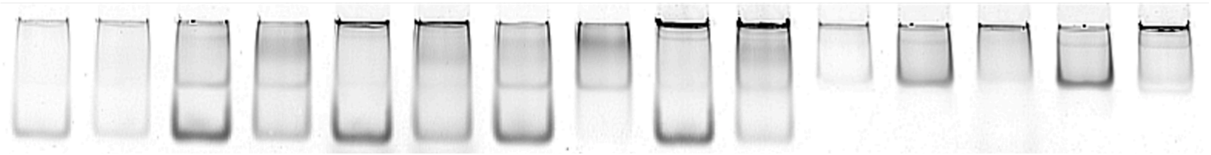

Supplement: Supplementary file 1 — SI [file 41598_2017_15361_MOESM1_ESM.pdf]
